# Lice, rodents, and many hopes: a rare disease in a young refugee

**DOI:** 10.1186/s13054-017-1666-5

**Published:** 2017-04-03

**Authors:** Salvatore L. Cutuli, Gennaro De Pascale, Teresa Spanu, Antonio M. Dell’Anna, Maria G. Bocci, Federico Pallavicini, Fabiola Mancini, Alessandra Ciervo, Massimo Antonelli

**Affiliations:** 1 Department of Anesthesiology and Intensive Care, Policlinico Universitario A. Gemelli Università Cattolica del Sacro Cuore, Largo A. Gemelli, 8 00168 Rome, Italy; 2 Department of Microbiology, Policlinico Universitario A. Gemelli Università Cattolica del Sacro Cuore, Largo A. Gemelli, 8 00168 Rome, Italy; 3 Institute of Infectious Diseases, Policlinico Universitario A. Gemelli Università Cattolica del Sacro Cuore, Largo A. Gemelli, 8 00168 Rome, Italy; 4 Department of Infectious, Parasitic and Immuno-mediated Diseases, Istituto Superiore di Sanità, Rome, Italy

**Keywords:** Migrants, *Borrelia recurrentis*, *Leptospira*, Borreliosis, Leptospirosis

Migrants from countries with scarce resources represent an increasing worldwide phenomenon providing a daily challenge for governments and humanitarian organizations [1, 2].

A teenage refugee from East Africa was admitted to our intensive care unit (ICU) with acute respiratory distress syndrome (ARDS), hypotension, and jaundice. Nits were present on her scalp and she had no relevant past medical history. She arrived in Italy after travelling for 7 months under poor hygienic conditions.

ARDS was managed with protective mechanical ventilation (tidal volume 350 ml, plateau pressure 28 cmH_2_O), high positive end-expiratory pressure (15 cmH_2_O), neuromuscular blocking agents, prone positioning, and inhaled nitric oxide. Septic shock and sepsis-induced cardiac dysfunction required administration of high doses of norepinephrire (0.8 μg/kg/min) and dobutamine (8 μg/kg/min). Continuous renal replacement therapy (CRRT) was started for acute kidney injury. Laboratory findings were relevant for anemia, low platelet count, altered blood coagulation, and high procalcitonin. Microbiological tests were performed before the administration of piperacillin-tazobactam and levofloxacin along with the application of pyrethrins foam.

In the differential diagnosis we evaluated epatotropic viruses, *Legionella* species, miliary tuberculois, intestinal parasites, *Schistosoma Haematobium*, *Rickettsia* species, *Leptospira* species, *Borrelia* species, *Leishmania* species, and *Malaria* species related infections.

On day 3, the blood and urine samples were positive on real-time polymerase chain reaction (PCR) [3, 4] for *Leptospira* spp. (Fig. 1a) and *Borrelia recurrentis* (only in the blood sample; Fig. 1b). Antibiotic therapy with 100 mg doxycycline every 12 h and 2 g ceftriaxone every 12 h was started, leading to a progressive improvement of the patient’s clinical status. On day 21 she was moved to the infectious disease ward, and 10 days later she ran away the hospital and has never come back for clinic follow-up.

**Fig. 1:**
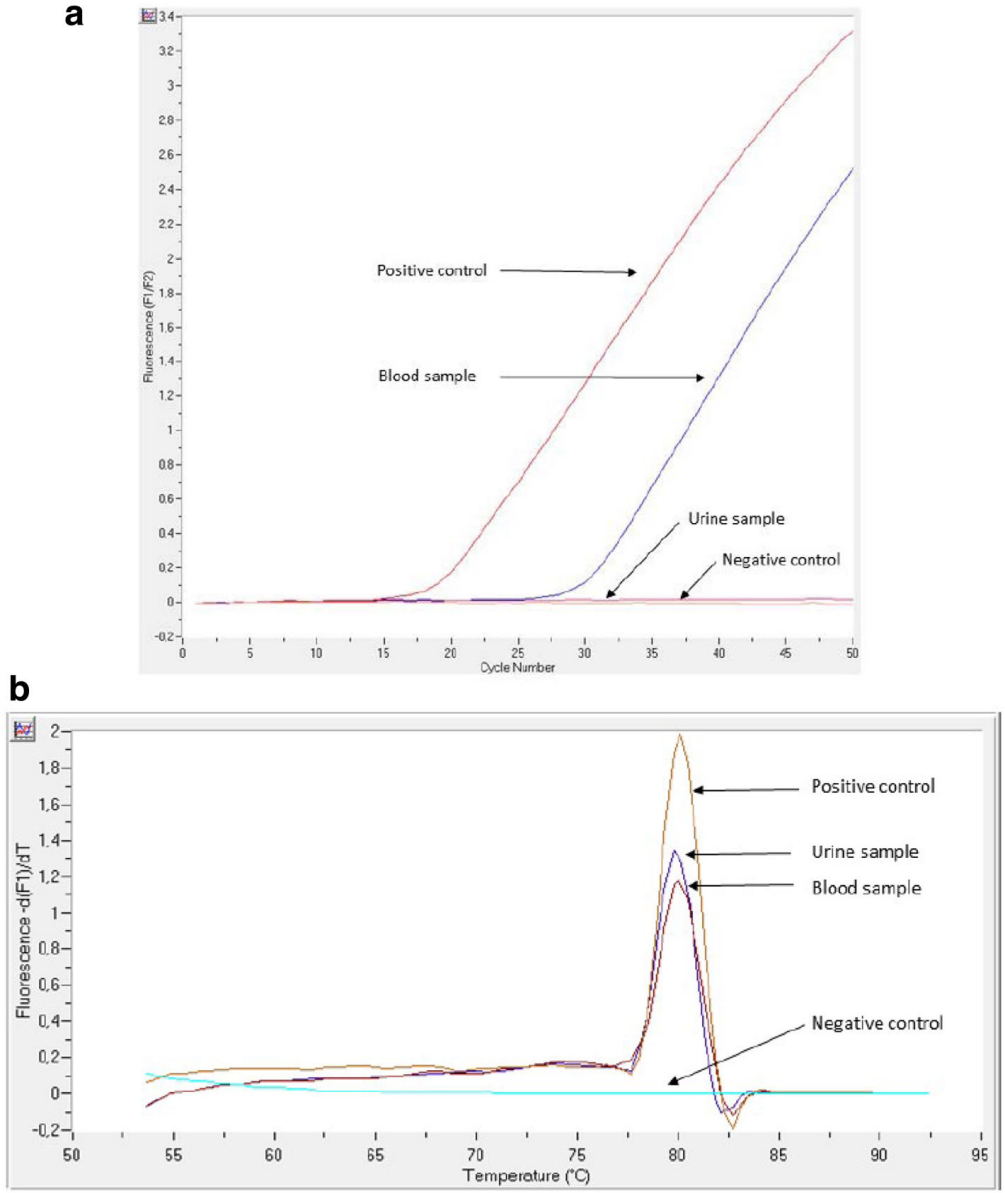
**a** A specific real-time PCR, able to detect the *secY* house keeping gene, was used to detect pathogenic *Leptospira* species in blood and urine. **b** The detection of the louse-borne relapsing fever (LBRF) agent was carried out by a species-specific real-time PCR, targeting an internal region of the *B. recurrentis/B. duttonii recN* gene. The nucleotide sequence analysis of the 16S rRNA multispacer sequence typing (MST) was utilized as a molecular tool for the microbial genotyping of LBRF

*Borrelia recurrentis* infection is a louse-borne disease and *Leptospirosis* is a rat-borne zoonosis, both endemic in areas characterized by a low hygiene condition. This is the first case of life-threatening *Borrelia recurrentis* and *Leptospira* species co-infection [1, 2, 5]. Spirochetosis-related disease is considered a rare pathology in nonendemic areas whereby the infection might be underdiagnosed. Delay in diagnosis and therapy may lead to dangerous outbreaks in refugees camps leading to severe clinical pictures in infected subjects.

Our patient ran away from the hospital without completing the path of care, being afraid of being repatriated. Indeed, even though we are able provide such patients with all the latest technologies, we cannot completely care for them without taking into account their social, psychological, and human needs.

## Abbreviations

ARDS: Acute respiratory distress syndrome
CRRT: Continuous renal replacement therapy
ICU: Intensive care unit
PCR: Polymerase chain reaction
